# A Facile Microwave Hydrothermal Method for Fabricating SnO_2_@C/Graphene Composite With Enhanced Lithium Ion Storage Properties

**DOI:** 10.3389/fchem.2022.895749

**Published:** 2022-06-01

**Authors:** Li-Lai Liu, Ming-Yang Li, Yi-Han Sun, Xue-Ying Yang, Min-Xuan Ma, Hui Wang, Mao-Zhong An

**Affiliations:** ^1^ College of Environmental and Chemical Engineering, Heilongjiang University of Science and Technology, Harbin, China; ^2^ School of Chemical Engineering and Technology, Harbin Institute of Technology, Harbin, China; ^3^ Baotailong New Materials Co.,Ltd., Jixi, China

**Keywords:** SnO_2_@C/graphene, porous structure, electrochemical performance, lithium-ion batteries, microwave hydrothermal

## Abstract

SnO_2_@C/graphene ternary composite material has been prepared via a double-layer modified strategy of carbon layer and graphene sheets. The size, dispersity, and coating layer of SnO_2_@C are uniform. The SnO_2_@C/graphene has a typical porous structure. The discharge and charge capacities of the initial cycle for SnO_2_@C/graphene are 2,210 mAh g^−1^ and 1,285 mAh g^−1^, respectively, at a current density of 1,000 mA g^−1^. The Coulombic efficiency is 58.60%. The reversible specific capacity of the SnO_2_@C/graphene anode is 955 mAh g^−1^ after 300 cycles. The average reversible specific capacity still maintains 572 mAh g^−1^ even at the high current density of 5 A g^−1^. In addition, cyclic voltammetry (CV) and electrochemical impedance spectroscopy (EIS) are performed to further investigate the prepared SnO_2_@C/graphene composite material by a microwave hydrothermal method. As a result, SnO_2_@C/graphene has demonstrated a better electrochemical performance.

## Introduction

Lithium-ion batteries (LIBs) have been used to operate various electric devices over the last decade, and the capacity, rate performance, and cycle stability of LIBs rely directly on the electrode materials (Yu et al., 2020; Cheng et al., 2021). Among various anode materials, graphite is the major anode material of commercial LIBs, which has excellent rate performances due to its small inner resistances, fast electron, and lithium-ion transport kinetics (Liu et al., 2016; Gong et al., 2020). However, its applications in energy storage are limited due to the low-theoretical capacity (372 mAh g^−1^), considering the high energy density requirements from LIBs (Chang et al., 2018; Liu et al., 2020). In order to meet the growing demand and expand the range of applications for high-performance LIBs, the development of advanced anode materials is crucial, such as Sn-based oxides (Hu et al., 2021; Kuriganova et al., 2016; Das et al., 2016), Si-based oxides (He et al., 2017; Xiang et al., 2017), Sb-based oxides (Zhou et al., 2019), Fe-based oxides (Li H. et al., 2021; Li et al., 2017), Co-based oxides (Li Q. et al., 2021), Ti-based oxides (Huang et al., 2018), transition metal sulfides (Zhang et al., 2021; Xiao et al., 2021), and others (Gao et al., 2020; Karahan et al., 2019), can provide their high theoretical lithium storage capacity and attract much attention.

In recent years, SnO_2_ has been investigated as potential anode candidates for LIBs due to its chemical stability, abundance, low-cost, environmentally friendly, and high theoretical specific capacity (782 mAh g^−1^) (Zhang et al., 2016; Zhou et al., 2019). However, SnO_2_ displays particle aggregation and large volume changes (>300%) during lithiation/delithiation, and these defects lead to the pulverization of active materials, breakage of solid electrolyte interphase (SEI), rapid capacity fading, poor cycling performance, and poor rate performance (Li et al., 2016; Ding et al., 2018; Ming et al., 2018). Carbon materials are considered to be an effective strategy to overcome the aforementioned shortcomings. Because carbon cushions the volume change, prevents the active matter from exposing to the electrolyte and enhances the electrical conductivity of the overall electrode and is easy to control and highly effective, resulting in significantly improved electrochemical performance (Zhu et al., 2017; Zhao et al., 2019; Wang et al., 2020). In particular, graphene is a good choice due to its unique merits of large aspect ratios, large surface areas, high electrical conductivity, and excellent mechanical properties (Zhang et al., 2020).

To surmount these limitations, in this report, SnO_2_@C/graphene composite has been synthesized by a microwave hydrothermal method. The features of the method are quick heating, easily controlled pressure and temperature, high yield rate, and good homogeneity. Our results indicate that SnO_2_@C/graphene can significantly improve the LIB properties, which is due to the 3D network structure of SnO_2_@C/graphene and the synergistic effect of graphene and SnO_2_@C. This material would overcome the drawbacks of SnO_2_ and show a promising candidate as an anode for LIBs.

## Experimental

### Preparation of Graphite Oxide and Graphene Oxide

The expanded graphite was synthesized from natural large flakes of graphite by the method reported in our previous work (Liu et al., 2015). The graphene oxide solution was synthesized by a modification to Hummer’s method. Briefly, 2 g expanded graphite was added to a 500-ml beaker, followed by 150 ml concentrated sulfuric acid; the beaker was then placed in an ice-water bath and stirred for 10 min. Potassium permanganate (8 g) was added slowly and stirred for 30 min, and then the beaker was placed in a water bath at 35 uC and stirred with a mechanical stirrer for 24 h. Deionized water (100 ml) was added to the beaker at 98 uC and stirred for 10 min. Then, 40 ml H_2_O_2_ (30%) was added to the mixture and continuously stirred for 1 h. The mixture was filtered and washed with 10% HCl and 1% H_2_O_2_ solutions until the pH was 5. Graphite oxide of 1 mg mL^−1^ as a precursor material was exfoliated by high-power ultrasonication for 30 min.

### Preparation of SnO_2_@C

First, 200 ml of 0.04 mol·L^-1^ SnCl4 solution was prepared, and 0.1 g of carbon spheres was dissolved in 20 ml of deionized water. Then, 8 ml SnCl_4_ solution was added slowly. The obtained product was sonicated for about another 30 min to assure the homogeneous dispersion. The reaction was transferred into a sealed Teflon-lined stainless steel autoclave and conducted for hydrothermal treatment at 180°C for 8 h. In order to improve the carbonization degree of SnO_2_@C composites, the prepared samples were heat-treated for 120 min at 500°C under nitrogen gas.

### Preparation of SnO_2_@C/Graphene Composite

The prepared SnO2@C material was added to 120 ml of 1 mg ml^-1^ graphene oxide aqueous solution, followed by ultrasonic treatment for 30 min. The mixed solution was transferred to high pressure Teflon vessels of the microwave reaction system (Anton-Paar Synthos 3000). The system power, temperature, pressure, and reaction time were 1200 W, 180°C, 2.0 MPa, and 60 min, respectively. The black as-synthesized product was cleaned several times by centrifugation with deionized water and dried at 80°C.

### Sample Characterization

The materials were characterized using a scanning electron microscope (SEM, Quanta 200F), transmission electron microscopy (TEM, FEI Tecnai G2 F20), and X-ray diffraction (XRD, Bruker D8 Advance with Cu Kα radiation) operated at 40 kV and 40 mA; Raman spectra (Renishaw RM-1000) were recorded using a plus laser Raman spectrometer with an excitation laser beam wavelength of 514.5 nm; FTIR analysis was carried out using pressed KBr disks in the range of 4,000–400 cm^−1^ using a PerkinElmer spectrometer.

### Electrochemical Measurements

The electrochemical measurements were carried out using CR2025 coin-type cells. The working electrode was prepared by the method reported in our previous work, which was coating slurry consisting of active material, PVDF (polyvinylidene fluoride), and acetylene black with a weight ratio of 80:10:10 in NMP (N-methyl-pyrrolidone) solvent (Liu et al., 2015).

## Results

### Microstructural Characterization

#### X-Ray Diffraction Analysis

**FIGURE 1:**
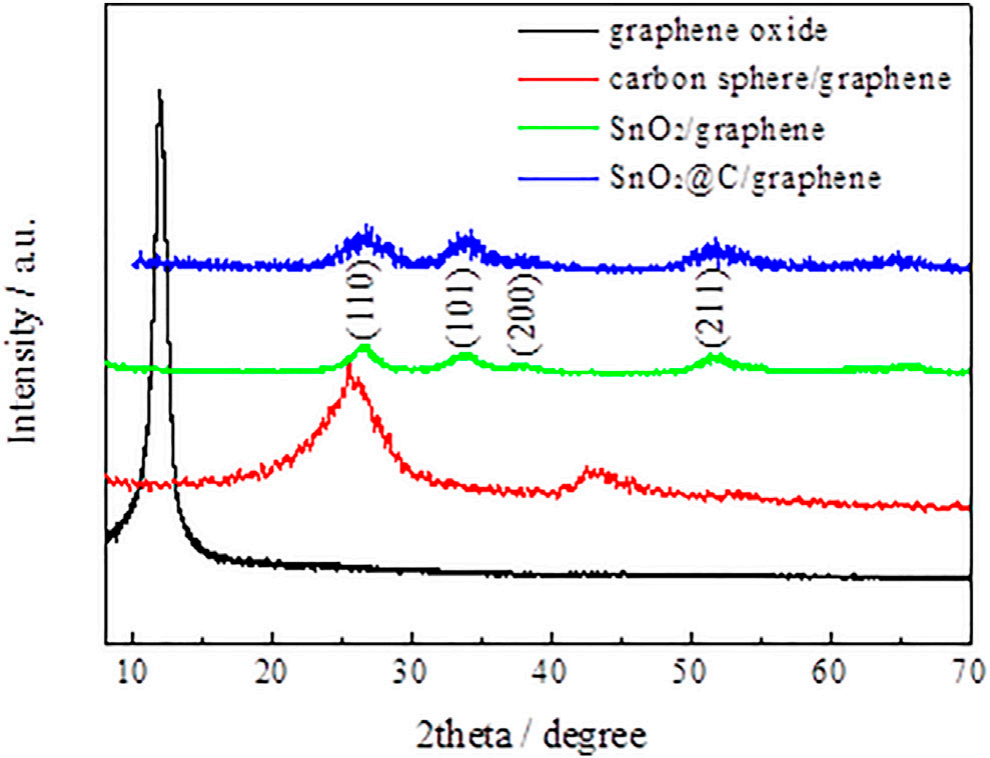
XRD patterns of graphene oxide, carbon spheres/graphene, SnO_2_/graphene, and SnO_2_@C/graphene.

Figure 1 shows XRD patterns of graphene oxide, carbon spheres/graphene, SnO_2_/graphene, and SnO_2_@C/graphene. The XRD pattern of SnO_2_@C/graphene is similar to that of SnO_2_/graphene. The obvious diffraction peaks at 26.7°, 33.9°, 38.2°, and 51.6° are associated with (110), (101), (200), and (211) of crystalline SnO_2_, respectively (JCPDS card no. 41–1445). The XRD patterns of SnO_2_@C/graphene are different from carbon spheres/graphene composites. There are no diffraction peaks of graphene near 26° and 43°, indicating that the surface of graphene is uniformly loaded with the SnO_2_@C composite material, and the diffraction peaks of graphene coincide with SnO_2_ particles or are covered by the diffraction peak of the SnO_2_@C composite material.

### Scanning Electron Microscope Analysis

**FIGURE 2:**
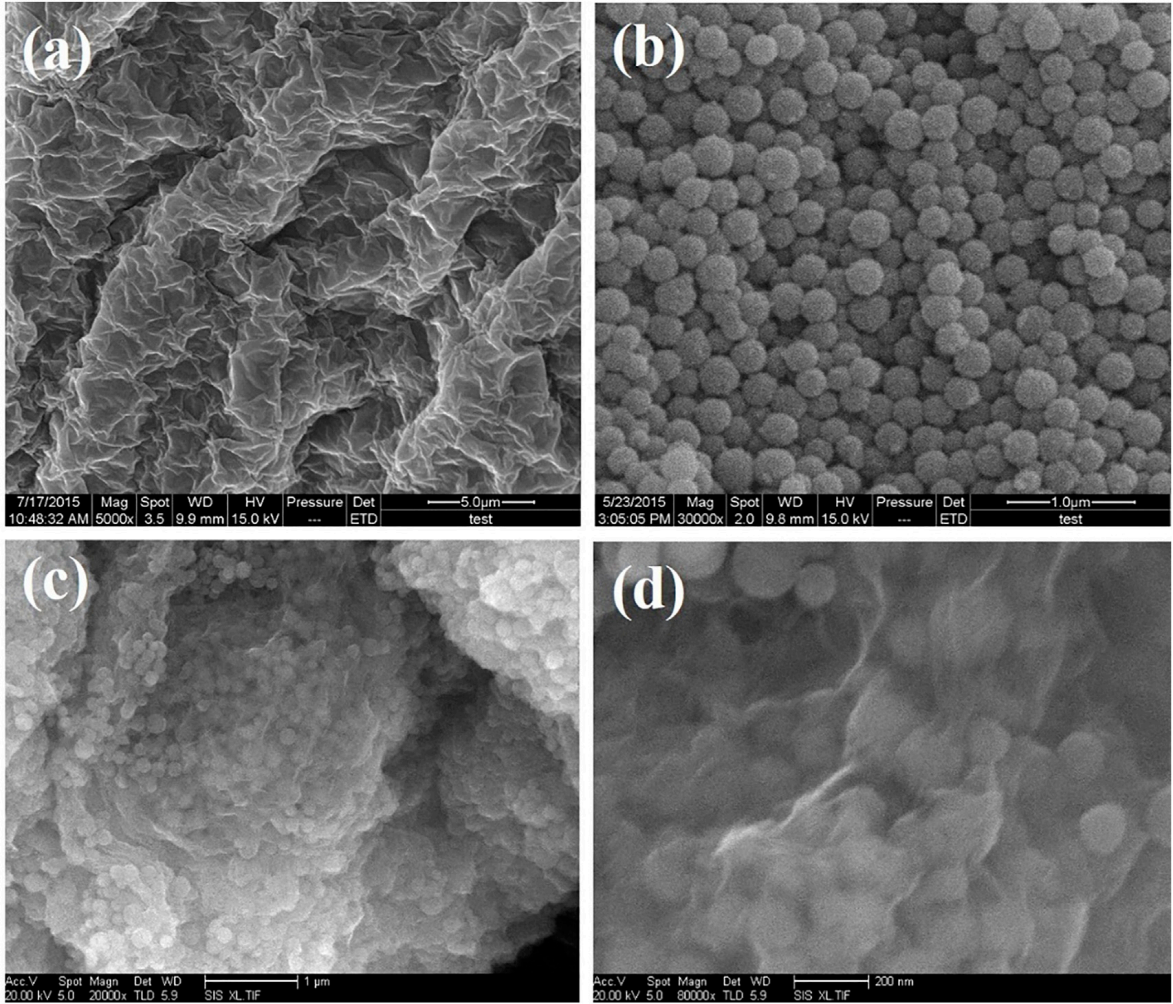
SEM images of graphene oxide **(A)**, SnO_2_@C **(B)**, and SnO_2_@C/graphene **(C,D)** samples.

Figure 2 shows SEM images of graphene oxide, SnO_2_@C, and SnO_2_@C/graphene samples. Figure 2A is a typical SEM image of graphite oxide, showing the layered platelets that are composed of curled and wrinkled graphene oxide sheets. Also, it is obvious that the graphene oxide sheets are agglomerated and overlapped due to no ultrasonic treatments. Figure 2B is a typical SEM image of SnO_2_@C composite, showing that the diameter of SnO_2_@C composite is 100–120 nm. Figures 2C,D are SEM images of SnO_2_@C/graphene which is prepared by the microwave hydrothermal method. It can be seen from the Figures 2C,D that the SnO_2_@C material is coated with graphene and distributed uniformly on the surface of graphene. SnO_2_@C/graphene composite with a three-dimensional structure is synthesized successfully by a microwave hydrothermal method.

### FTIR Analysis

**FIGURE 3:**
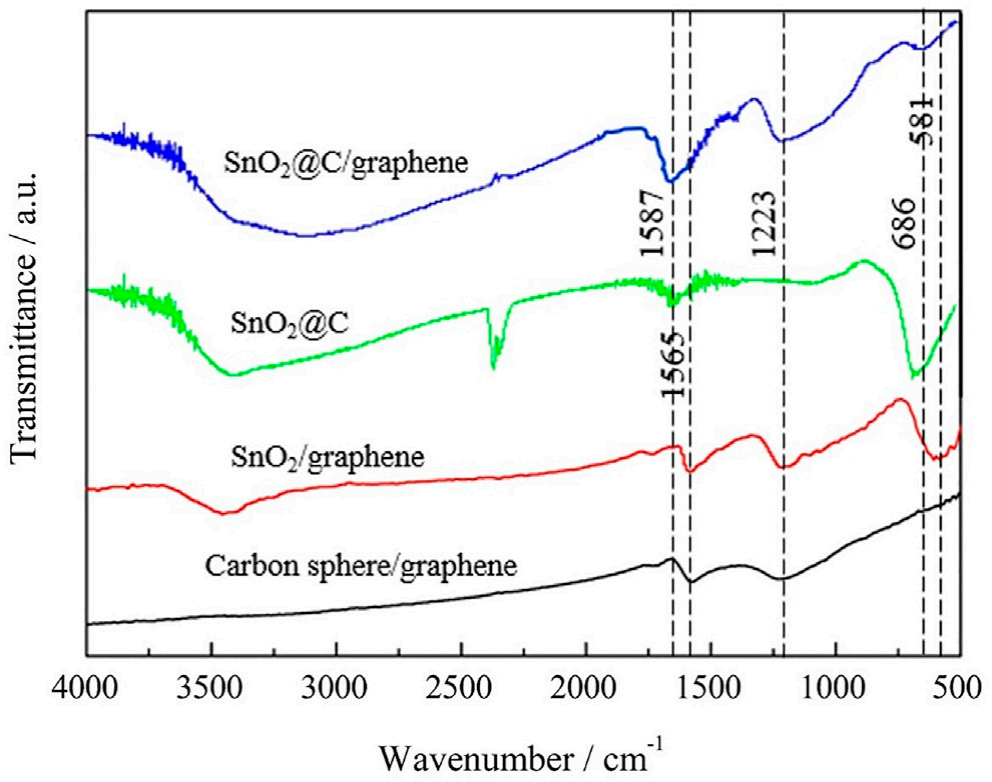
FTIR spectra of carbon spheres/graphene, SnO_2_/graphene, SnO_2_@C, and SnO_2_@C/graphene.

Figure 3 shows FTIR spectra of carbon spheres/graphene, SnO_2_/graphene, SnO_2_@C, and SnO_2_@C/graphene. The broad absorption peak in the range of 3000–3500 cm^−1^ can be attributed to O–H stretching vibrations. The absorption peaks at 1,565 cm^−1^ of carbon spheres/graphene and SnO_2_/graphene and the absorption peaks at 1,587 cm^−1^ of SnO_2_@C and SnO_2_@C/graphene are all attributed to skeletal vibrations of C=C. The absorption peak at 1,223 cm^−1^ is attributed to C–O stretching vibrations, indicating that a small amount of oxygen-containing functional groups still exists in the composite. The absorption peak of SnO_2_/graphene is detected at 581 cm^−1^ that corresponds to Sn–O–Sn antisymmetric vibrations. The absorption peaks of SnO_2_@C and SnO_2_@C/graphene are detected at 686 cm^−1^ that also correspond to Sn–O–Sn antisymmetric vibrations. From the FTIR spectra of SnO_2_@C/graphene, we know that the characteristic absorption bands of these oxide groups decrease obviously or almost disappear, indicating that the SnO_2_@C composite can be further carbonized and graphene oxide is deoxidized to produce graphene under the high temperature and high pressure of the microwave hydrothermal method (Jiang et al., 2014; Liu et al., 2014).

### Raman Analysis

**FIGURE 4:**
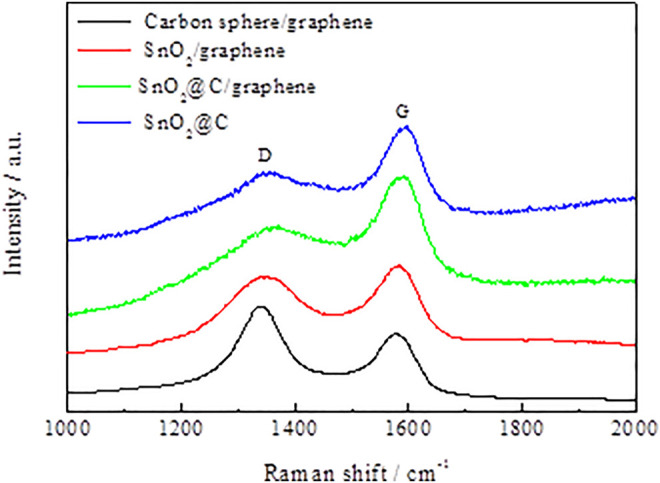
Raman spectra of carbon spheres/graphene, SnO_2_/graphene, SnO_2_@C, and SnO_2_@C/graphene.

Figure 4 shows Raman spectra of carbon spheres/graphene, SnO_2_/graphene, SnO_2_@C, and SnO_2_@C/graphene. It can be seen from the figure that in the Raman spectra of carbon spheres/graphene and SnO_2_/graphene, obvious peaks are present at 1,346 cm^−1^ (D band) and 1,581 cm^−1^ (G band), respectively. However, in the Raman spectra of SnO_2_@C and SnO_2_@C/graphene, the peaks at 1,357 cm^−1^ (D band) and 1,592 cm^−1^(G band), respectively, move up by 11 cm^−1^. The enhancement can be attributed to the presence of SnO_2_ nanoparticles, which are loaded on the graphene sheets and the carbon spheres (Zhang et al., 2012). The D band is an indication of defects associated with vacancies, grain boundaries, and amorphous carbon species (Wang et al., 2009); the G band corresponds to the E2g mode of graphite, which is related to the vibration of sp2-bonded carbon atoms in a two-dimensional hexagonal lattice (Zhang et al., 2012; Zuo et al., 2018). The intensity ratio (ID/IG) of the D band to the G band is related to the extent of disorder degree and average size of the sp2 domains (Ferrari and Robertson, 2000; Feng et al., 2020). The ID/IG value of the SnO_2_@C is 0.62, and the ID/IG value of the SnO_2_@C/graphene is 0.83, indicating that the disordered structure of the SnO_2_@C/graphene is obviously enhanced.

**FIGURE 5:**
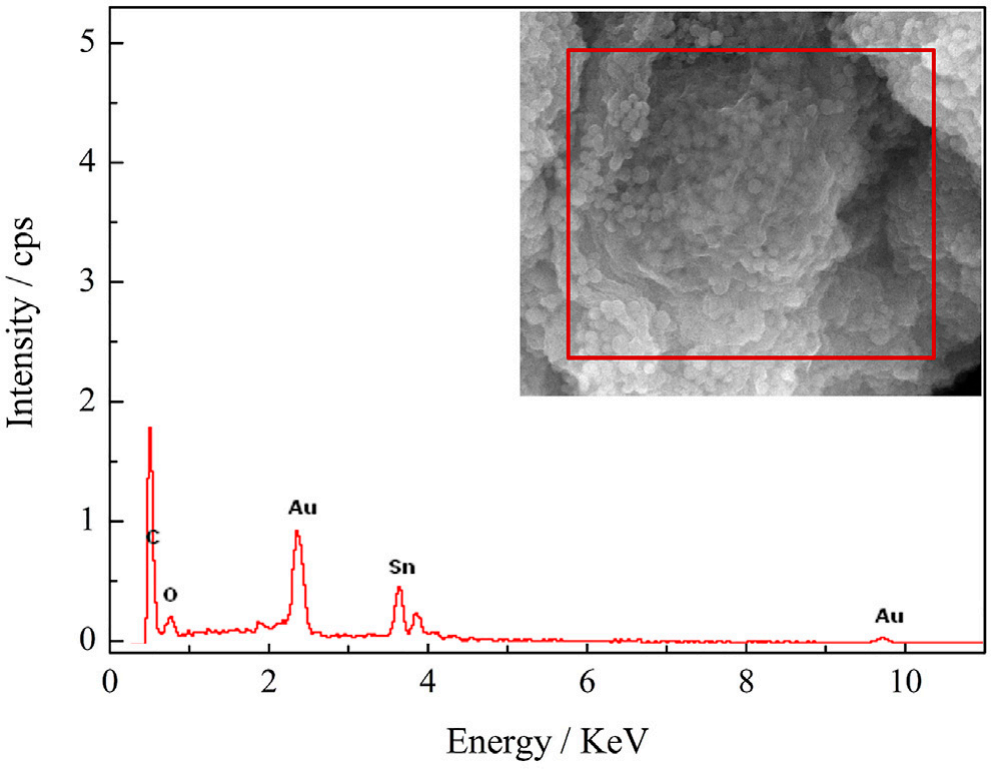
EDS image of SnO_2_@C/graphene.

Figure 5 shows the EDS image of SnO_2_@C/graphene, which reveals the presence of carbon, oxygen, and tin. The element content of SnO_2_@C is shown in Table 1. The element content of SnO_2_@C/graphene is shown in Table 2. Due to the addition of graphene, the mass fraction of C increases from 38.76% of SnO_2_@C to 56.67%, and the mass fractions of O and Sn are 10.10% and 33.23%, respectively. The element content of C increases, and the element content of O and Sn all decreases, indicating that the sheet structure in SEM images is graphene sheets.

**TABLE 1:** Element content of SnO_2_@C.

Element	Mass fraction (%)	Atomic ratio (%)
C	38.76	72.52
O	13.09	18.37
Sn	48.15	9.11
Matrix	Correction	ZAF

**TABLE 2:** Element content of SnO_2_@C/graphene

Element	Mass fraction(%)	Atomic ratio(%)
C	56.67	83.81
O	10.10	11.21
Sn	33.23	4.97
Matrix	Correction	ZAF

### Electrochemical Property Analysis

**FIGURE 6:**
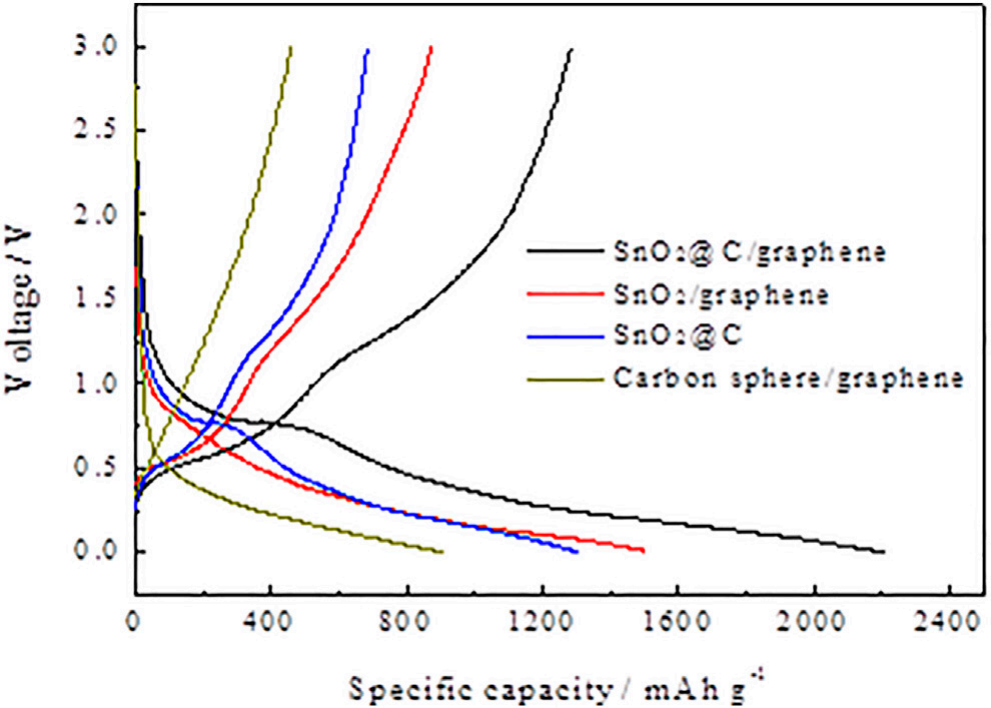
First charge–discharge profiles of carbon spheres/graphene, SnO_2_/graphene, SnO_2_@C, and SnO_2_@C/graphene.

Figure 6 shows the first charge–discharge profiles of carbon spheres/graphene, SnO_2_/graphene, SnO_2_@C, and SnO_2_@C/graphene at a current density of 1,000 mA g^−1^ with a voltage range of 0.01–3 V. The first discharge- and charge-specific capacities of SnO_2_@C/graphene are 2,210 and 1,285 mAh g^−1^, respectively, and the Coulomb efficiencies of the first cycle is 58.60%. Also, the first discharge capacity of SnO_2_@C/graphene is higher than that of carbon ball/graphene, SnO_2_@C, and SnO_2_/graphene materials. The reason is that the 3D network structure of SnO_2_@C/graphene has a higher specific surface area and more defects, which can provide more activated sites for Li storage.

**FIGURE 7:**
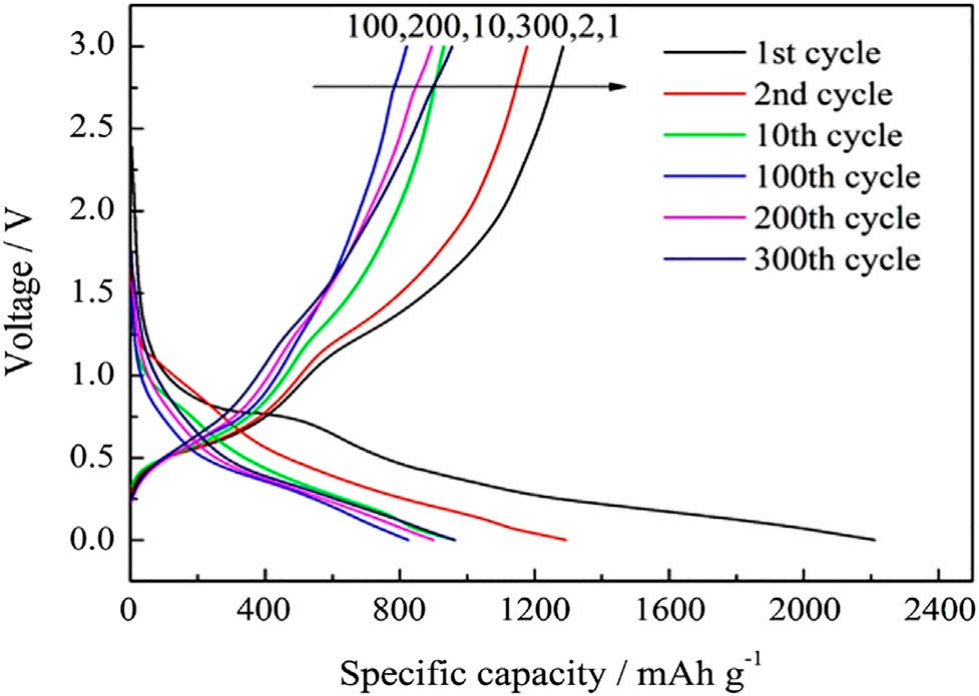
Charge–discharge profiles of SnO_2_@C/graphene cycled for the 1st, 2nd, 10th, 100th, 200th, and 300th cycle at a current density of 1,000 mA g^−1^.

Figure 7 shows the charge–discharge profiles of SnO2@C/graphene cycled for the first, second, 10th, 100th, 200th, and 300th cycle at a current density of 1,000 mA g^−1^. The first discharge occurs due to the irreversible capacity loss, and the charge–discharge curves are very similar to the 10th cycle, indicating that the structure of the SnO_2_@C/graphene electrode is relatively stable during the process of charge–discharge, and the cycling performance is better at a large current density.

**FIGURE 8:**
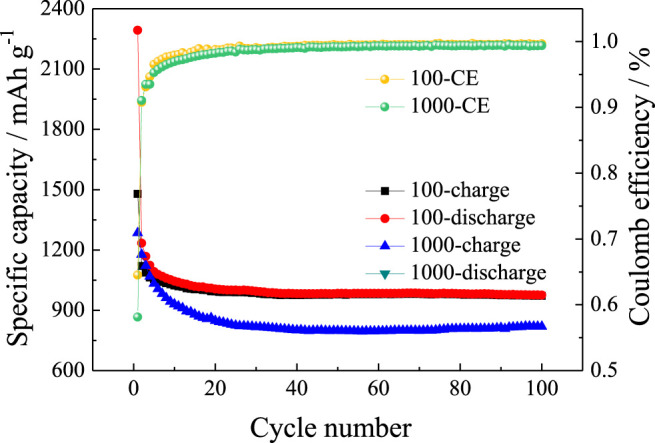
Cycle performances of SnO_2_@C/graphene at different current densities.

Figure 8 shows cycle performances of SnO2@C/graphene at current densities of 100 mA g^−1^ and 1,000 mA g^−1^. At current densities of 100 mA g^−1^, the reversible specific capacity of SnO_2_@C/graphene is 971 mAh g^−1^ after 100 cycles with the Coulomb efficiencies of 99.6% and the capacity retention of 65.7%. At current densities of 1,000 mA g^−1^, the reversible specific capacity of SnO_2_@C/graphene is 820 mAh g^−1^ after 100 cycles with the Coulomb efficiencies of 99.4% and the capacity retention of 63.8%. The result shows that SnO_2_@C/graphene has a better cycling performance at a large current density.

**FIGURE 9:**
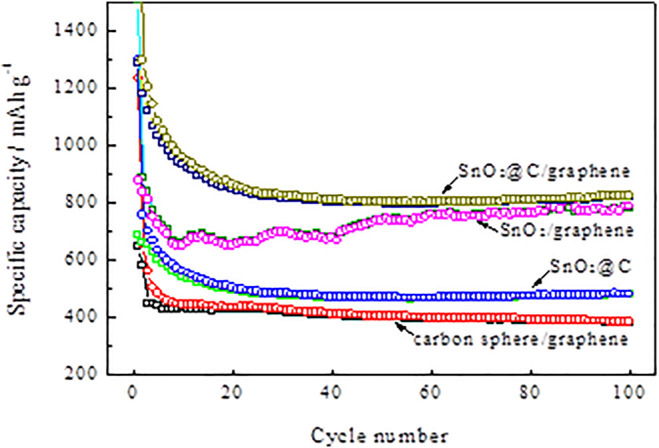
Cycle performances of carbon spheres/graphene, SnO_2_/graphene, SnO_2_@C, and SnO_2_@C/graphene at a current density of 1,000 mA g^−1^.

Figure 9 shows the cycle performances of carbon spheres/graphene, SnO_2_/graphene, SnO_2_@C, and SnO_2_@C/graphene at a current density of 1,000 mA g^−1^. The SnO_2_@C/graphene electrode shows better cycling performance. The reversible specific capacity of SnO_2_@C/graphene (820 mAh g^−1^) after 100 cycles is much higher than that of carbon spheres/graphene (386 mAh g^−1^), SnO_2_@C (480 mAh g^−1^), and SnO_2_/graphene (778 mAh g^−1^). Also, the reversible specific capacity of SnO_2_@C/graphene shows a trend of rising with the cycle of charge–discharge.

**FIGURE 10:**
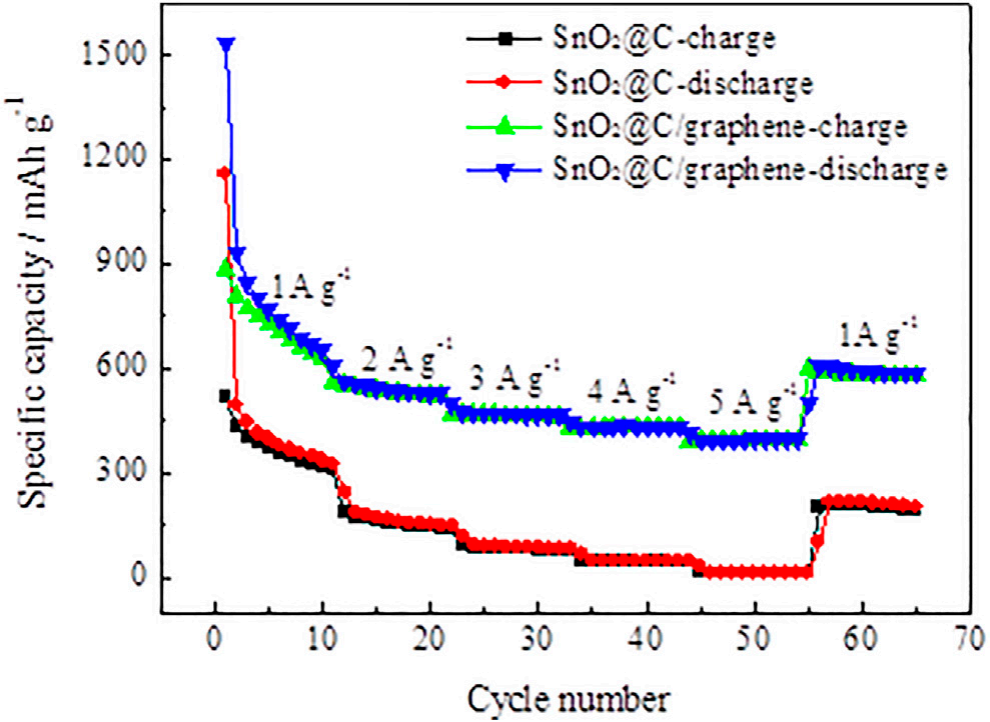
Rate capability of SnO_2_@C and SnO_2_@C/graphene.

The SnO_2_@C and SnO_2_@C/graphene electrode are cycled at different current densities ranging from 1 A g^−1^ to 5 A g^−1^ and then reversed back to the current density of 1 A g^−1^. Figure 10 shows the rate capability of SnO_2_@C and SnO_2_@C/graphene. The average reversible specific capacities of the SnO_2_@C electrode material at different current densities are 634 mAh g^−1^, 265 mAh g^−1^, 140 mAh g^−1^, 76 mAh g^−1^, and 22 mAh g^−1^. When the current density is reduced to 1 A g^−1^, the average reversible specific capacity is 277 mAh g^−1^ after 10 cycles, and the capacity retention of SnO_2_@C is 43.7%. Compared with SnO_2_@C, the SnO_2_@C/graphene composites show a much better rate performance. The average reversible specific capacities of the SnO_2_@C/graphene electrode material at current densities of 1 A g^−1^, 2 A g^−1^, 3 A g^−1^, 4 A g^−1^, and 5 A g^−1^ are 1,052 mAh g^−1^, 779 mAh g^−1^, 676 mAh g^−1^, 625 mAh g^−1^, and 572 mAh g^−1^, respectively. When the current density is reduced to 1 A g^−1^, the average reversible specific capacity is 788 mAh g^−1^ after 10 cycles, and the capacity retention of SnO_2_@C/graphene is 74.9%. It can be seen that the rate performance of SnO_2_@C/graphene is much greater than that of SnO_2_@C. This indicates that the presence of graphene and the synergistic effect of graphene and SnO_2_@C play an important role, which improves the rate performance of electrode material.

**FIGURE 11:**
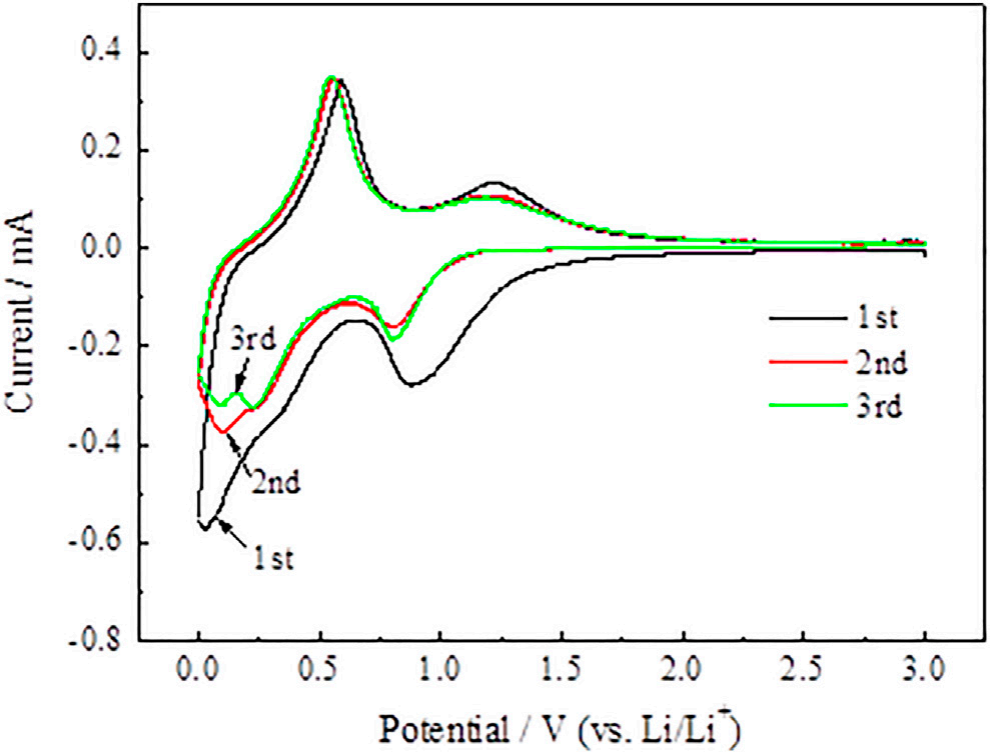
CV curves of SnO_2_@C composite.

**FIGURE 12:**
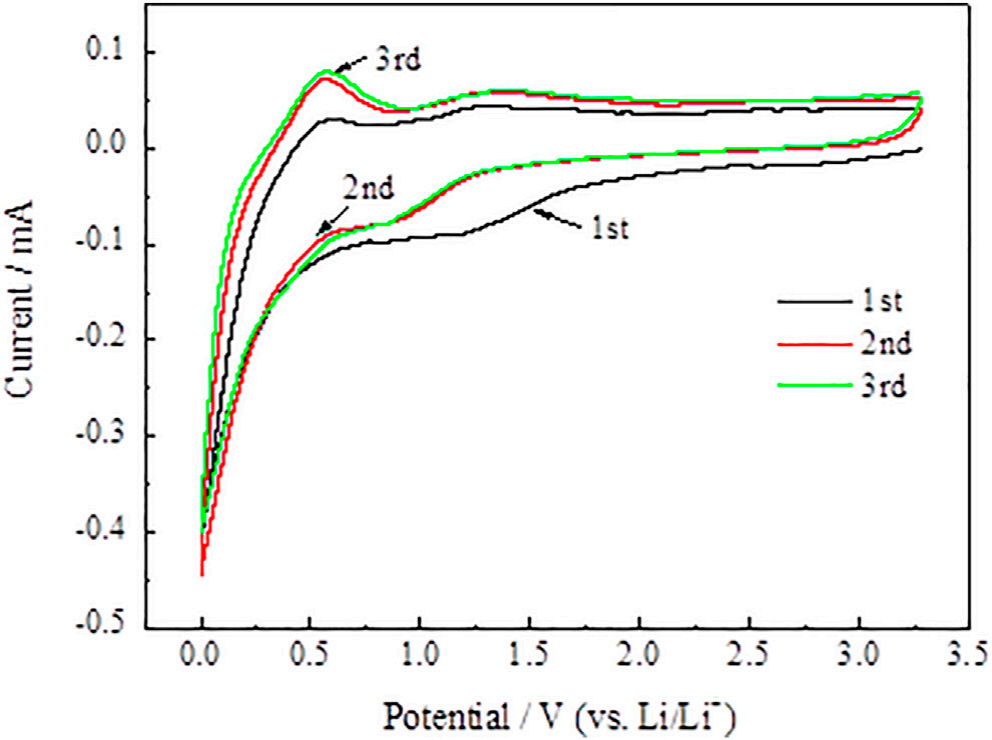
CV curves of SnO_2_@C/graphene.

The synergy mechanism between SnO2@C and SnO2@C/graphene is explored with cyclic voltammetry. Figure 11 and Figure 12 represent the CV of SnO_2_@C and SnO_2_@C/graphene for the cycles 1 to 3 in the voltage range of 0.01–3.00 V at 0.1 mV·s^−1^. As shown in Figure 11, an obvious peak around 0.85 V is found in the cathodic polarization curve of the first cycle, which can be ascribed to the formation of a solid–electrolyte interface (SEI) layer on the surface of the electrode from the reduction of electrolyte decomposition and the reduction of SnO_2_ (Wang et al., 2017; Narsimulu et al., 2018). The cathode peak appears in the potential ranging from 0.01 to 0.30 V, which is mainly due to the formation of LixSn and Li + intercalation into nano carbon spheres (Kim et al., 2012). Then, it can be seen from the anodic polarization curve of the first cycle, the oxidation peaks at about 0.22 and 0.75 V are mainly attributed to the dealloying of LixSn, which is corresponding to the opposite process of Li–Sn alloying (Thomas and Mohan Rao, 2014; Shi et al., 2017). The oxidation peak at about 1.25 V is ascribed to the reversible reaction of Sn generated by the SnO_2_@C electrode.

In Figure 12, compared with the SnO_2_@C electrode material, a low cathode peak of the SnO_2_@C/graphene electrode material is present at about 1.25 V after the first cycle. It indicates that the SnO_2_@C/graphene consumes much less Li than SnO_2_@C and has less irreversible capacity. Therefore, the first Coulomb efficiency of the SnO_2_@C/graphene electrode material is greatly improved.

**FIGURE 13:**
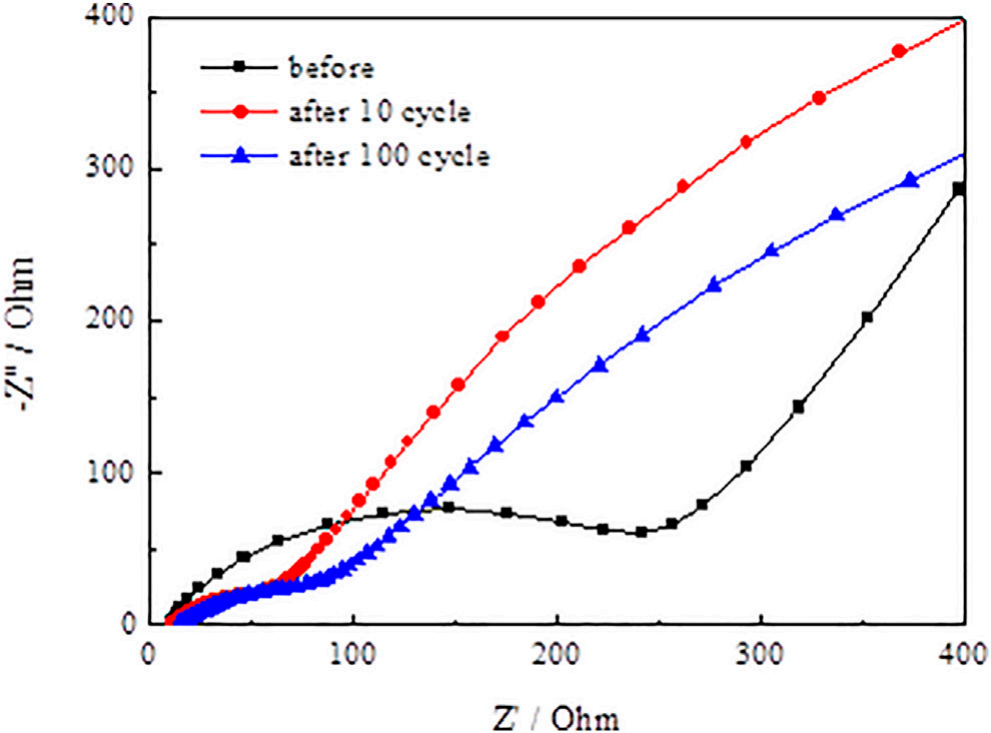
Nyquist plots of SnO_2_@C.

**FIGURE 14:**
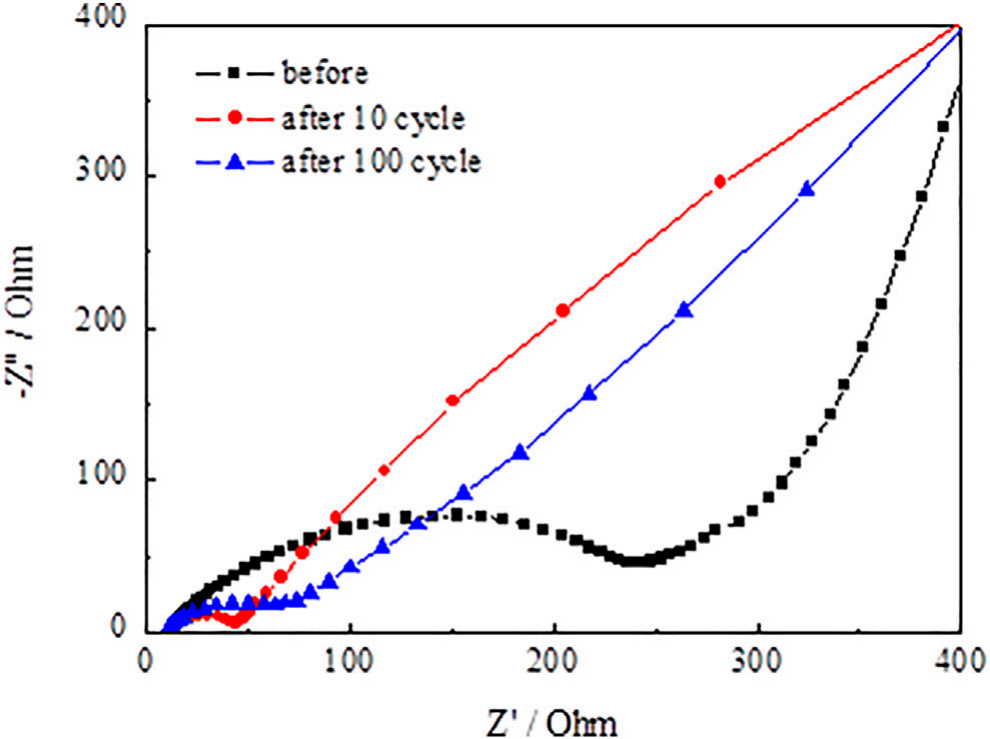
Nyquist plots of SnO_2_@C/graphene.

Figure 13 and Figure 14 present the Nyquist plots of SnO_2_@C and SnO_2_@C/graphene by applying an AC voltage of 5 mV in the frequency range of 0.01–100 kHz. In Figure 13 and Figure 14, it can be seen that the semicircle radius of the Nyquist curve of SnO_2_@C/graphene in the high–middle frequency region is significantly smaller than that of SnO_2_@C after the 10th cycle and 100th cycle. This result indicates that the Rct of SnO_2_@C/graphene is obviously less than that of SnO_2_@C and the addition of graphene can improve the conductivity of electrode material.

**FIGURE 15:**
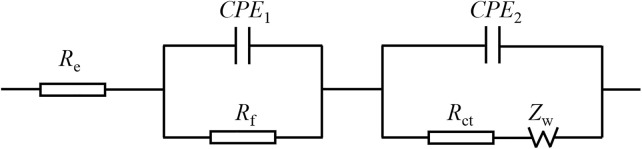
Randles equivalent circuit for SnO_2_@C and SnO_2_@C/graphene.

Figure 15 presents the Randles equivalent circuit for SnO_2_@C and SnO_2_@C/graphene. Re represents the resistance imposed by the electrolyte, electrode, and diaphragm. Rf and CPE1 represent the resistance of the solid electrolyte interface (SEI) film adjacent to the electrode and capacity. Rct and CPE2 represent the charge transfer resistance and electric double layer capacitor. Zw represents Warburg impedance associated with the diffusion of the lithium ions into the electrode material. Table 3 is the kinetic parameters of the SnO_2_@C and SnO_2_@C/graphene electrodes. The electrochemical impedance of the SnO_2_@C and SnO_2_@C/graphene is 297.6 Ω cm^2^ and 257.5 Ω cm^2^, respectively, before charging and discharging. The electrochemical impedance of the SnO_2_@C and SnO_2_@C/graphene is 109.3 Ω cm^2^ and 45.8 Ω cm^2^ after 10 cycles, respectively. The electrochemical impedance of the SnO_2_@C and SnO_2_@C/graphene is 150.3 Ω cm^2^ and 82.4 Ω cm^2^ after 100 cycles, respectively. This result further shows that the addition of graphene can improve the conductivity of the electrode material. Therefore, compared with SnO_2_@C, the reversible specific capacity and rate performance of the SnO_2_@C/graphene electrode material are greatly improved.

**TABLE 3:** Kinetic parameters of the SnO_2_@C and SnO_2_@C/graphene electrodes.

Electrode	SnO_2_@C	SnO_2_@C/Graphene
0 cycle	297.6 Ω cm^2^	257.5 Ω cm^2^
After 10 cycles	109.3 Ω cm^2^	45.8 Ω cm^2^
After 100 cycles	150.3 Ω cm^2^	82.4 Ω cm^2^

## Conclusion

The SnO_2_@C/graphene composite is synthesized successfully by a microwave hydrothermal method for lithium-ion batteries. Owing to the merit of the composite structure, the first discharge- and charge-specific capacities of SnO_2_@C/graphene are 2,210 and 1,285 mAh g^−1^, respectively; the Coulomb efficiency is 58.60%. The reversible specific capacity of SnO_2_@C/graphene is 820 mAh g^−1^ after 100 cycles. The average reversible specific capacities of the SnO_2_@C/graphene electrode material even at a current density of 5 A g^−1^ is 572 mAh g^−1^. The SnO_2_@C/graphene electrode material has better electrochemical lithium storage performance, which is mainly ascribed to the 3D network structure of SnO_2_@C/graphene and the synergistic effect of graphene and SnO_2_@C. The carbon nanospheres with better dispersion and homogeneity structure not only stabilize SnO_2_ nanoparticles but also buffer the volume expansion of SnO_2_ nanoparticles. Graphene can prevent agglomeration of SnO_2_@C, improve the electrical conductivity, and also buffer the volume expansion of SnO_2_ nanoparticles. Therefore, graphene can improve electrochemical lithium storage performance of the SnO_2_@C/graphene electrode material.

## Data Availability

The original contributions presented in the study are included in the article/Supplementary Material; further inquiries can be directed to the corresponding authors.
